# Internal Jugular Vein Injury by Fishbone Ingestion

**DOI:** 10.1155/2020/9182379

**Published:** 2020-06-18

**Authors:** Armin Amirian, Hamed Ghoddusi Johari, Mohamadreza Karoobi, Reza Shahriarirad, Keivan Ranjbar

**Affiliations:** ^1^Thoracic and Vascular Surgery Research Center, Shiraz University of Medical Sciences, Shiraz, Iran; ^2^Trauma Research Center, Vascular Surgery Department, Shiraz University of Medical Sciences, Shiraz, Iran; ^3^Student Research Committee, Shiraz University of Medical Sciences, Shiraz, Iran

## Abstract

Fishbone ingestion is a common occurrence in the Middle East countries. We present a patient with a unique complication of fishbone ingestion. A 65-year-old woman presented with left-sided neck pain and swelling since 5 days before admission. A linear foreign body with horizontal orientation was seen in CT scan at the superior part of the pharynx along with a collection around it which caused a laceration on the medial aspect of internal jugular vein and thrombosis inside the internal jugular vein.

## 1. Introduction

In countries where fish is often consumed, fishbones are among the most frequently encountered foreign bodies in the upper gastrointestinal tract. Esophageal foreign body impaction usually presents acutely, especially in adults, who normally have a clear history of ingestion. Adult patients may have diffuse chest pain, a vague foreign body sensation, a sensation of choking, or neck or throat pain [1]. The risk of complications varies from perforation of the gastrointestinal tract to hepatic abscess [2, 3]. In this case report, we present a unique complication of fishbone ingestion which led to internal jugular vein injury.

## 2. Case Presentation

**Figure 1:**
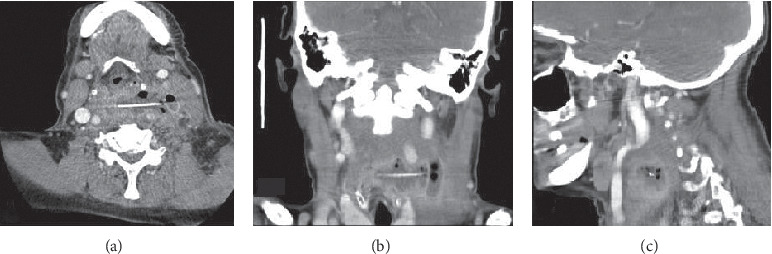
Fishbone foreign body revealed in CT scan. (a) Axial view, (b) coronal view, and (c) sagittal view of the foreign body with horizontal orientation in the superior part of the pharynx.

A 65-year-old woman presented with left-sided neck pain and swelling since 5 days prior to admission. She reported progressive dysphagia during this period after a history of grilled fish ingestion. There was no pertinent past medical and family history, and she denied any drug or substance abuse. Physical examination revealed low-grade fever (T: 38°C oral) and mild tachycardia (HR: 95 bpm). There was marked stiffness of the neck with an 8 × 8 cm erythematous bulging anterior to left sternocleidomastoid muscle at the level of the thyroid cartilage. A spiral neck CT with IV contrast was done. A linear foreign body with horizontal orientation was seen in the superior part of the pharynx along with a 40 × 20 mm collection around it (Figure 1). There was also thrombosis with an air bubble in the left internal jugular vein, and the distal end of the fishbone was adjacent to the left internal jugular vein. Due to the high risk of endoscopic removal, we decided to proceed to surgery for better exposure and also effective drainage of infection. Via a classic incision anterior to the left sternocleidomastoid muscle, hypopharynx and cervical esophagus were explored and the carotid sheath was opened. A 4 cm sharp fishbone was found impacted transversely into hypopharynx at the level of the thyroid cartilage and thrombosed internal jugular vein. After meticulous removal of fishbone, a 3 × 3 mm laceration was found on the medial aspect of the internal jugular vein. Systemic heparinization was done just before ligation of internal jugular vein with a stat dose of 5000 unit unfractionated heparin administered intravenously then internal jugular vein was ligated proximal and distal to the site of injury. Perforation of the pharynx was repaired in two layers. Drainage of abscess cavities and debridement of necrotic tissues were done, and samples were sent for culture. A Penrose drain was inserted in paravertebral region, the skin was closed with separate stitches, and systemic heparinization was ceased. The postoperative course was uneventful, and liquid diet was started on the 4th postoperative day. In such settings, ultrasound and barium esophagram are useful tools to evaluate postoperative condition of the patients. Since serious intracranial sequelae after unilateral ligation of internal jugular vein are extremely rare, routine ultrasound evaluation after surgery is not advised unless signs of intracranial hypertension occur; furthermore, preoperative CT scan of the patient had documented patency of contralateral internal jugular vein, therefore after a normal barium esophagram, the patient was discharged on the 5th postoperative day.

## 3. Discussion

A foreign body in the upper gastrointestinal tract happens occasionally as food is ingested, and in several cases, it can cause irritation and pain. Among gastrointestinal foreign bodies, 80%–90% pass spontaneously. Among those, less than 1% will need surgical intervention [2]. Fishbone is the most frequent cause of foreign body ingestion in adults, especially in Asia, compared to meat in Western countries [4].

Ingestion of fishbone can cause mucosal ulceration and inflammation. It could lead to esophageal perforation, mediastinitis, pneumothorax [5], gastric perforation leading to gastric outlet obstruction [6], and many other catastrophic consequences. Bleeding and perforation are more common in fishbone foreign body ingestion than other ingested foreign bodies [7]. Fishbones usually lodge at the physiological strictures of the esophagus. The esophagus has three areas of physiological narrowing: the upper esophageal sphincter, eminence of the aortic arch, and the lower esophageal sphincter. Also, pathologies, such as stenosis, will create another site. The upper esophageal sphincter is the most common lodging site of the fishbone foreign body [4, 8, 9].

The diagnosis of the fishbone foreign body is based on the patient's history and symptoms. A physical examination should evaluate the patient's general condition and assess signs of any complications [10]. The symptoms of esophageal foreign body diseases are foreign body sensation, sore throat, dysphagia, odynophagia, retrosternal pain, retching, and vomiting [11, 12]. Patients can generally confirm the ingestion and localize the irritation. However, the area of discomfort often does not represent the site of impaction but patients who are able to lateralize the presumptive foreign body within the cervical region then the object is likely to be above the cricopharyngeus [13]. Radiographic study of the neck, chest, and abdomen is necessary in order to assess the presence, location, size, configuration, and the number of ingested objects. It also helps to detect the foreign body induced complications [10, 14, 15].

The most common complication regarding foreign body ingestion in adults is a retropharyngeal abscess. However, in the fishbone foreign body, esophageal penetration or perforation occurs in more than 50% of cases [9].

Upper esophageal and oropharyngeal fishbone foreign bodies can cause soft tissue infection and other complications including fistula formation and carotid arterial injury [16, 17]. Studies indicated that the risk of complications increases with a longer duration of impaction (>24 hours) [18] and bone type [9] and length (>3 cm) [19].

Fishbones tend to migrate and extrude after perforating the esophagus to nearby structures [17]. The major concern is the penetration of major vascular structures. The role of plain radiography and CT scan in the assessment of foreign body ingestion has been investigated in previous studies and some guidelines consider these tools to be more useful than others such as sonography [20–23]; however, radiological visualization in plain radiography is dependent on radiopacity of the object. CT scan is useful for detecting complications such as perforations, and it has been reported to have a higher sensitivity in foreign body detection in comparison to plain radiography of lesser opaque objects, especially when they are surrounded by air [20].

The temporal and spatial resolutions of sonography also might offer hope for visualizing suspected nonopaque foreign objects located superficially; however, it might not be effective in localizing those located deep and inside the air-filled cavities [24, 25].

In patient follow-up, sonography is indicated when intracranial hypertension is detected. Since serious intracranial sequelae after unilateral ligation of internal jugular vein are extremely rare, routine ultrasound evaluation after surgery in an uneventful postoperative patient is not advised [26].

## 4. Conclusions

Fishbone ingestion can cause complications from retropharyngeal abscess to esophageal perforation and vascular injuries. Based on our case report, we recommend the CT scan imaging in patients with suspected esophageal sharp foreign body impaction with consideration of advanced complications such as the migration of the fishbone from the esophagus to the internal jugular vein, which lead to thrombosis in this vein.

## Data Availability

Data of the case report are available upon request to the corresponding author via mail.
